# The genome sequence of the setaceous Hebrew character, *Xestia c-nigrum*, (Linnaeus, 1758)

**DOI:** 10.12688/wellcomeopenres.18608.1

**Published:** 2022-12-06

**Authors:** Gavin R. Broad, Douglas Boyes, Natural History Museum Genome Acquisition Lab, University of Oxford and Wytham Woods Genome Acquisition Lab, Darwin Tree of Life Barcoding collective, Wellcome Sanger Institute Tree of Life programme, Wellcome Sanger Institute Scientific Operations: DNA Pipelines collective, Tree of Life Core Informatics collective, Darwin Tree of Life Consortium

**Affiliations:** 1 Department of Life Sciences, Natural History Museum, London, UK; 2 UK Centre for Ecology and Hydrology, Wallingford, Oxfordshire, UK

**Keywords:** Xestia c-nigrum, setaceous Hebrew character, genome sequence, chromosomal, Lepidoptera

## Abstract

We present a genome assembly from an individual male *Xestia c-nigrum* (the setaceous Hebrew character; Arthropoda; Insecta; Lepidoptera; Noctuidae). The genome sequence is 760 megabases in span. Most of the assembly is scaffolded into 31 chromosomal pseudomolecules, including the assembled Z sex chromosome. The mitochondrial genome has also been assembled and is 15.3 kilobases in length.

## Species taxonomy

Eukaryota; Metazoa; Ecdysozoa; Arthropoda; Hexapoda; Insecta; Pterygota; Neoptera; Endopterygota; Lepidoptera; Glossata; Ditrysia; Noctuoidea; Noctuidae; Noctuinae; Noctuini; *Xestia*; *Xestia c-nigrum* (Linnaeus, 1758) (NCBI:txid987431).

## Background

Known to most British lepidopterists as setaceous Hebrew character, *Xestia c-nigrum* is generally referred to as the spotted cutworm in the pest control literature. The latter name is derived from the appearance of the caterpillar while ‘ *c-nigrum’* and ‘Hebrew character’ reference the distinctive black marking on the forewing.

*X. c-nigrum* is a familiar, widespread species across Asia, Europe and North America. In much of its range, including Britain, there are two generations in a year, the second usually much larger. The summer and autumn cohort might be larger due to increased survival (larvae of the spring cohort over-winter) and/or immigration from further south ( Clancy *et al.*, 2012). Larvae feed on a variety of herbaceous plants and over-winter as diapausing larvae, growing at a slower rate than non-diapausing larvae ( Honek, 1979). A granulovirus isolated from *X. c-nigrum* has been used to develop virus-based biopesticides against agricultural pest moths, *e.g.* ( Goto *et al.*, 2015).

This is the second whole genome sequence for a *Xestia* moth species, following that of *X. xanthographa* ( Boyes & Holland, 2022). The sequenced individual was collected at Hever Castle during a collecting trip for the Natural History Museum Darwin Tree of Life sampling team.

## Genome sequence report

**Figure 1.**
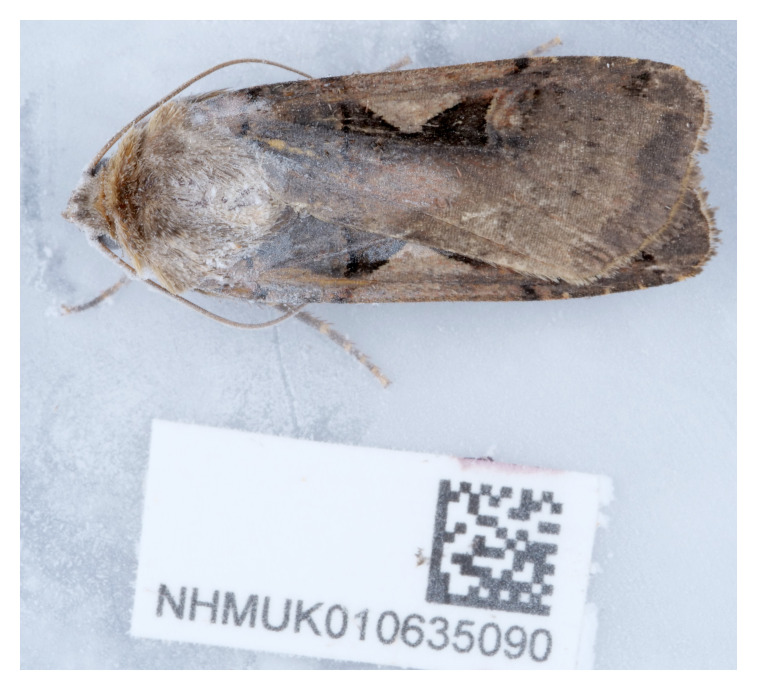
Image of the *Xestia c-nigrum* (ilXesCnig1) specimen used for genome sequencing.

The genome was sequenced from one male *X. c-nigrum* ( Figure 1) collected from Hever Castle, England, UK (latitude 51.188, longitude 0.12). A total of 35-fold coverage in Pacific Biosciences single-molecule HiFi long reads and 58-fold coverage in 10X Genomics read clouds were generated. Primary assembly contigs were scaffolded with chromosome conformation Hi-C data. Manual assembly curation corrected nine (9) missing/misjoins, reducing the assembly length by 0.03% and the scaffold number by 14%, and increasing the scaffold N50 by 0.1%.

**Figure 2.**
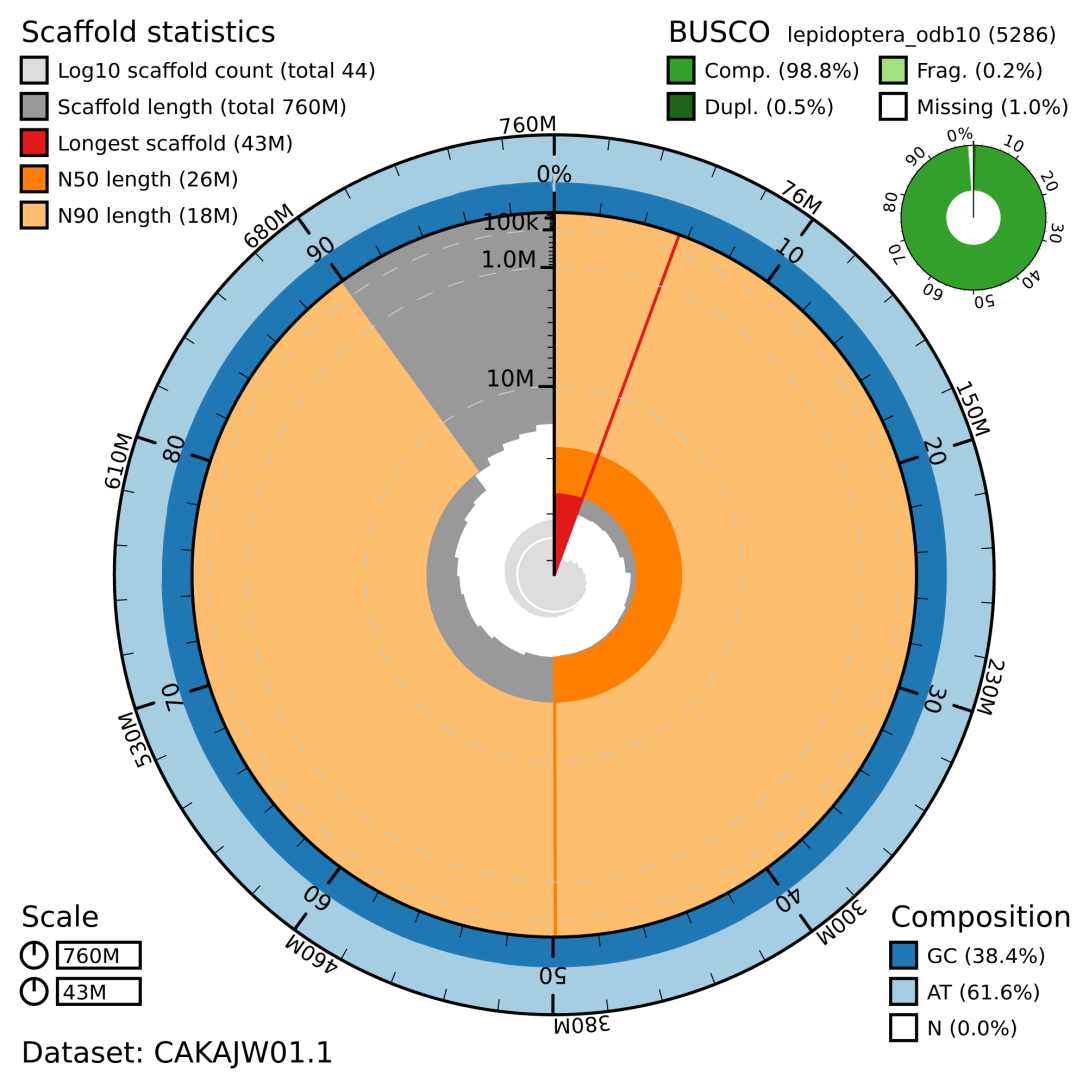
Genome assembly of *Xestia c-nigrum*, ilXesCnig1.1: metrics. The BlobToolKit Snailplot shows N50 metrics and BUSCO gene completeness. The main plot is divided into 1,000 size-ordered bins around the circumference with each bin representing 0.1% of the 760,318,956 bp assembly. The distribution of chromosome lengths is shown in dark grey with the plot radius scaled to the longest chromosome present in the assembly (43,326,432 bp, shown in red). Orange and pale-orange arcs show the N50 and N90 chromosome lengths (25,717,762 and 18,159,168 bp), respectively. The pale grey spiral shows the cumulative chromosome count on a log scale with white scale lines showing successive orders of magnitude. The blue and pale-blue area around the outside of the plot shows the distribution of GC, AT and N percentages in the same bins as the inner plot. A summary of complete, fragmented, duplicated and missing BUSCO genes in the lepidoptera_odb10 set is shown in the top right. An interactive version of this figure is available at https://blobtoolkit.genomehubs.org/view/ilXesCnig1.1/dataset/CAKAJW01.1/snail.

The final assembly has a total length of 760 Mb in 43 sequence scaffolds with a scaffold N50 of 25.7 Mb ( Table 1). Most of the assembly sequence (99.9%) was assigned to 31 chromosomal-level scaffolds confirmed by Hi-C data, representing 30 autosomes named in order of size and the Z chromosome ( Figure 2– Figure 5, Table 2). The assembly has a BUSCO 5.3.2 ( Manni *et al.*, 2021) completeness of 98.8% using the lepidoptera_odb10 reference set.

**Table 1.** Genome data for *Xestia c-nigrum* (ilXesCnig1.1).

Project accession data	
Assembly identifier	ilXesCnig1.1
Species	*Xestia c-nigrum*
Specimen	ilXesCnig1 (genome assembly, Hi-C), ilXesCnig2 (RNA-Seq)
NCBI taxonomy ID	987431
BioProject	PRJEB46327
BioSample ID	SAMEA8239458
Isolate information	Male, whole organism (ilXesCnig1); undescribed, whole organism (ilXesCnig2)
Raw data accessions	
PacificBiosciences SEQUEL II	ERR6939245, ERR6939246
10X Genomics Illumina	ERR6688565–ERR6688568
Hi-C Illumina	ERR6688569
PolyA RNA-Seq Illumina	ERR9435011
Genome assembly	
Assembly accession	GCA_916618015.1
*Accession of alternate haplotype*	GCA_916617455.1
Span (Mb)	760
Number of contigs	60
Contig N50 length (Mb)	25.0
Number of scaffolds	43
Scaffold N50 length (Mb)	25.7
Longest scaffold (Mb)	29.1
BUSCO * genome score	C:98.8%[S:98.2%,D:0.5%], F:0.2%,M:1.0%,n:5,286

* BUSCO scores based on the lepidoptera_odb1 BUSCO set using v5.3.2. C = complete [S = single copy, D = duplicated], F = fragmented, M = missing, n = number of orthologues in comparison. A full set of BUSCO scores is available at https://blobtoolkit.genomehubs.org/view/ilXesCnig1.1/dataset/CAKAJW01.1/busco.

**Figure 3.**
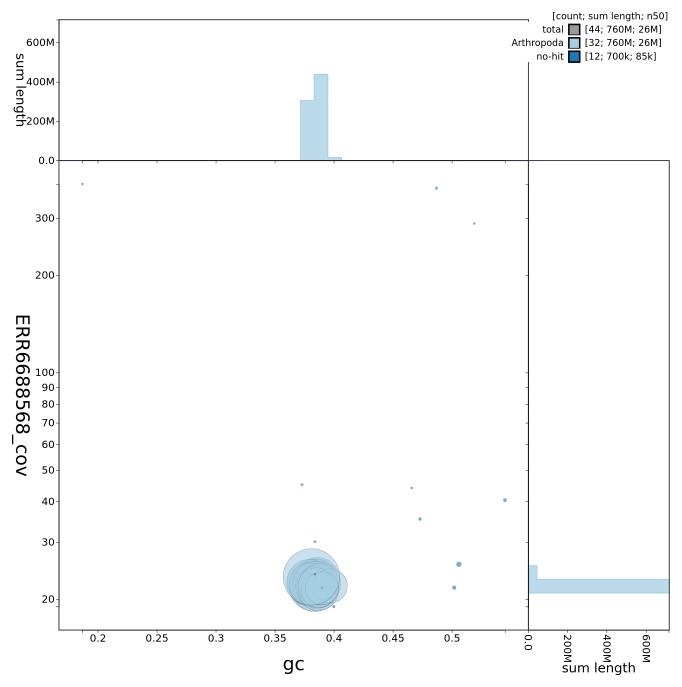
Genome assembly of *Xestia c-nigrum*, ilXesCnig1.1: GC coverage. BlobToolKit GC-coverage plot. Chromosomes are coloured by phylum. Circles are sized in proportion to chromosome length. Histograms show the distribution of chromosome length sum along each axis. An interactive version of this figure is available at https://blobtoolkit.genomehubs.org/view/ilXesCnig1.1/dataset/CAKAJW01.1/blob.

**Figure 4.**
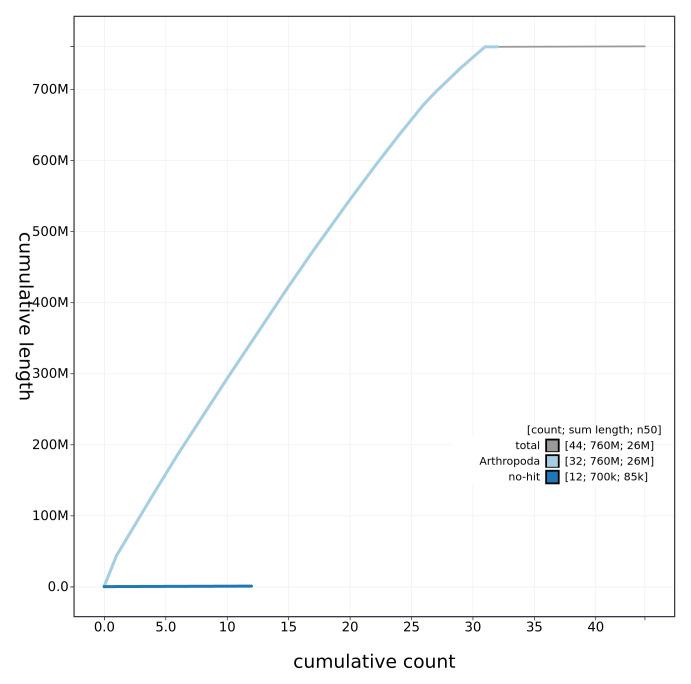
Genome assembly of *Xestia c-nigrum* (ilXesCnig1.1): cumulative sequence. BlobToolKit cumulative sequence plot. The grey line shows cumulative length for all chromosomes. Coloured lines show cumulative lengths of chromosomes assigned to each phylum using the buscogenes taxrule. An interactive version of this figure is available at https://blobtoolkit.genomehubs.org/view/ilXesCnig1.1/dataset/CAKAJW01.1/cumulative.

**Figure 5.**
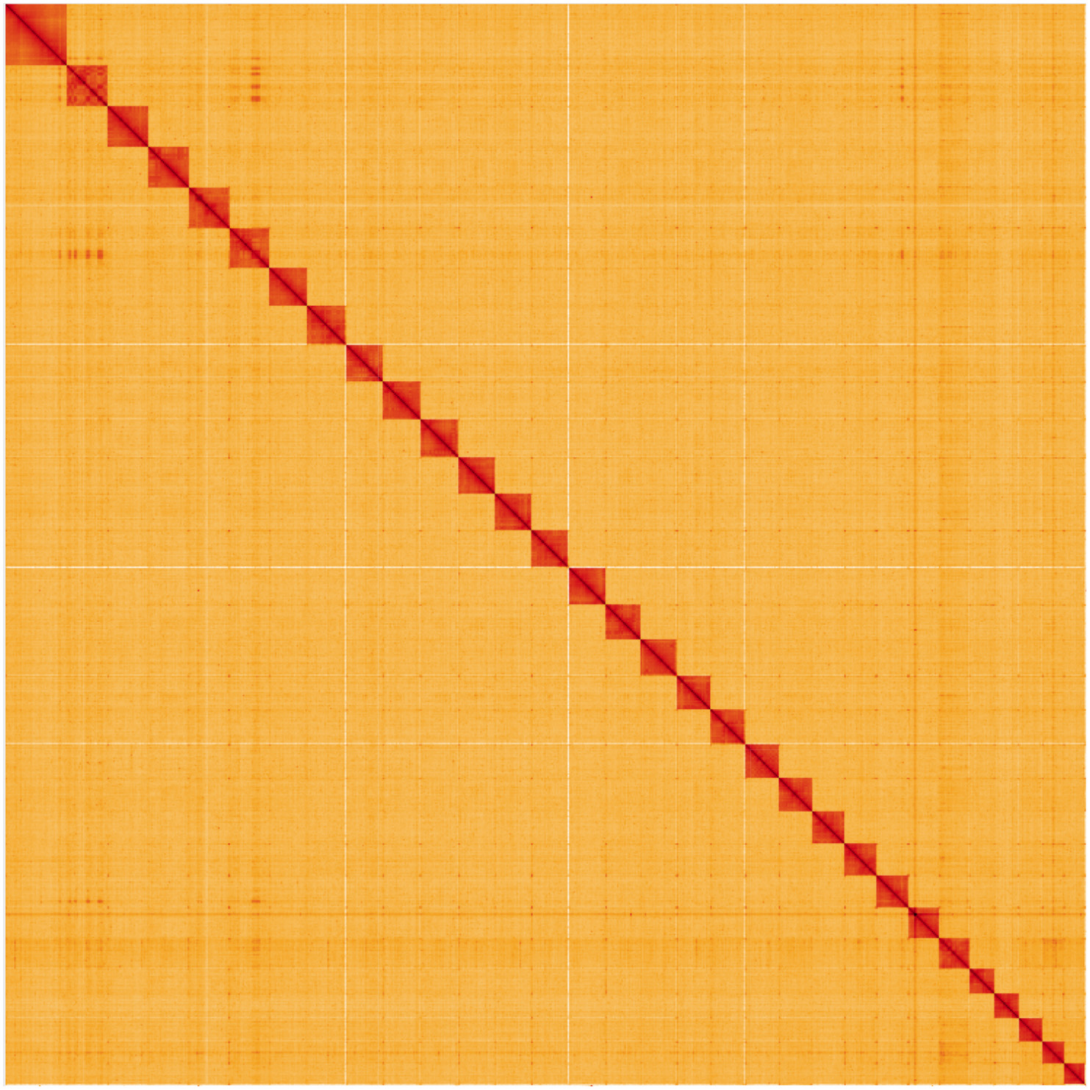
Genome assembly of *Xestia c-nigrum* (ilXesCnig1.1): Hi-C contact map. Hi-C contact map of the ilXesCnig1.1 assembly, visualised using HiGlass. Chromosomes are given in order of size from left to right and top to bottom. The interactive Hi-C map can be viewed at https://genome-note-higlass.tol.sanger.ac.uk/l/?d=CB8TqIaYQNqsG4GcF9YwLg.

**Table 2.** Chromosomal pseudomolecules in the genome assembly of *Xestia c-nigrum*, ilXesCnig1.

INSDC accession	Chromosome	Size (Mb)	GC%
OU745244.1	1	29.1	38.2
OU745245.1	2	28.86	38.4
OU745246.1	3	28.59	38.4
OU745247.1	4	28.48	38.3
OU745248.1	5	27.79	38.4
OU745249.1	6	26.8	38.3
OU745250.1	7	26.79	37.9
OU745251.1	8	26.44	38.6
OU745252.1	9	26.25	38.2
OU745253.1	10	26.15	38.1
OU745254.1	11	26.05	38.6
OU745255.1	12	26	38.5
OU745256.1	13	25.72	38.3
OU745257.1	14	25.7	37.9
OU745258.1	15	25.09	38.4
OU745259.1	16	25.04	38.5
OU745260.1	17	24.44	38.4
OU745261.1	18	23.84	38.5
OU745262.1	19	23.83	38.3
OU745263.1	20	23.45	38.6
OU745264.1	21	23.03	38
OU745265.1	22	22.35	38.7
OU745266.1	23	22.32	38.5
OU745267.1	24	21.62	38.9
OU745268.1	25	21.11	38.7
OU745269.1	26	18.16	38.5
OU745270.1	27	17.02	38.5
OU745271.1	28	16.11	38.7
OU745272.1	29	15.52	39.7
OU745273.1	30	14.63	39
OU745243.1	Z	43.33	38.1
OU745274.1	MT	0.02	19
-	unplaced	0.7	48

While not fully phased, the assembly deposited is of one haplotype. Contigs corresponding to the second haplotype have also been deposited.

## Methods

### Sample acquisition and nucleic acid extraction

A male *X. c-nigrum* (ilXesCnig1) was collected using a light trap and identified by Gavin Broad (Natural History Museum) from Hever Castle, United Kingdom (latitude 51.188, longitude 0.12). The sample was preserved on dry ice by Laura Sivess. A second *X. c nigrum* (ilXesCnig2) was collected using a light trap and identified by Douglas Boyes (Natural History Museum) from Wytham Woods, United Kingdom (latitude 51.772, longitude –1.338).

DNA was extracted from head and thorax tissue of ilXesCnig1 at the Wellcome Sanger Institute (WSI) Scientific Operations core using the Qiagen MagAttract HMW DNA kit, according to the manufacturer’s instructions. RNA was extracted from abdomen tissue of ilXesCnig2 in the Tree of Life Laboratory at the WSI using TRIzol, according to the manufacturer’s instructions. RNA was then eluted in 50 μl RNAse-free water and its concentration assessed using a Nanodrop spectrophotometer and Qubit Fluorometer using the Qubit RNA Broad-Range (BR) Assay kit. Analysis of the integrity of the RNA was done using Agilent RNA 6000 Pico Kit and Eukaryotic Total RNA assay.

### Sequencing

Pacific Biosciences HiFi circular consensus and 10X Genomics read cloud DNA sequencing libraries were constructed according to the manufacturers’ instructions. Poly(A) RNA-Seq libraries were constructed using the NEB Ultra II RNA Library Prep kit. DNA and RNA sequencing was performed by the Scientific Operations core at the WSI on Pacific Biosciences SEQUEL II (HiFi), Illumina HiSeq 4000 (RNA-Seq) and Illumina NovaSeq 6000 (10X) instruments. Hi-C data were also generated from head tissue of ilXesCnig1 using the Arima v2 kit and sequenced on the Illumina NovaSeq 6000 instrument.

### Genome assembly

Assembly was carried out with Hifiasm ( Cheng *et al.*, 2021) and haplotypic duplication was identified and removed with purge_dups ( Guan *et al.*, 2020). One round of polishing was performed by aligning 10X Genomics read data to the assembly with longranger align, calling variants with freebayes ( Garrison & Marth, 2012). The assembly was then scaffolded with Hi-C data ( Rao *et al.*, 2014) using SALSA2 ( Ghurye *et al.*, 2019). Manual curation ( Howe *et al.*, 2021) was performed using HiGlass ( Kerpedjiev *et al.*, 2018) and Pretext ( Harry, 2022). Chromosome-scale scaffolds confirmed by the Hi-C data have been named in order of size. The mitochondrial genome was assembled using MitoHiFi ( Uliano-Silva *et al.*, 2021), which performs annotation using MitoFinder ( Allio *et al.*, 2020). The genome was analysed and BUSCO scores were generated within the BlobToolKit environment ( Challis *et al.*, 2020). Table 3 contains a list of all software tool versions used, where appropriate.

**Table 3.** Software tools used.

Software tool	Version	Source
BlobToolKit	3.2.6	( Challis *et al.*, 2020)
freebayes	v1.3.1-17-gaa2ace8	( Garrison & Marth, 2012)
hifiasm	0.15.3	( Cheng *et al.*, 2021)
HiGlass	1.11.6	( Kerpedjiev *et al.*, 2018)
longranger	2.2.2	https://support.10xgenomics.com/genome-exome/software/pipelines/latest/advanced/other-pipelines
MitoHiFi	2.0	( Uliano-Silva *et al.*, 2021)
PretextView	0.2.x	https://github.com/wtsi-hpag/PretextView
purge_dups	1.2.3	( Guan *et al.*, 2020)
SALSA	2.2	( Ghurye *et al.*, 2019)

## Data Availability

European Nucleotide Archive: Xestia c-nigrum (spotted cutworm). Accession number PRJEB46327. https://identifiers.org/ena.embl/PRJEB46327 ( Wellcome Sanger Institute, 2022). The genome sequence is released openly for reuse. The *Xestia c-nigrum* genome sequencing initiative is part of the Darwin Tree of Life (DToL) project. All raw sequence data and the assembly have been deposited in INSDC databases. The genome will be annotated using available RNA-Seq data and presented through the Ensembl pipeline at the European Bioinformatics Institute. Raw data and assembly accession identifiers are reported in Table 1.

## Author information

Members of the Natural History Museum Genome Acquisition Lab are listed here: https://doi.org/10.5281/zenodo.4790042.

Members of the University of Oxford and Wytham Woods Genome Acquisition Lab are listed here: https://doi.org/10.5281/zenodo.4789928.

Members of the Darwin Tree of Life Barcoding collective are listed here: https://doi.org/10.5281/zenodo.4893703.

Members of the Wellcome Sanger Institute Tree of Life programme are listed here: https://doi.org/10.5281/zenodo.4783585.

Members of Wellcome Sanger Institute Scientific Operations: DNA Pipelines collective are listed here: https://doi.org/10.5281/zenodo.4790455.

Members of the Tree of Life Core Informatics collective are listed here: https://doi.org/10.5281/zenodo.5013541.

Members of the Darwin Tree of Life Consortium are listed here: https://doi.org/10.5281/zenodo.4783558.
