# Supplementary material for: The genome sequence of the setaceous Hebrew character, Xestia c-nigrum, (Linnaeus, 1758)
Source: Wellcome Open Res. 2022 Dec 6;7:295. [Version 1] doi: 10.12688/wellcomeopenres.18608.1 (PMC9971640; doi:10.12688/wellcomeopenres.18608.1)
Supplement: Peer review history

# Peer review: Reviewer response for version 1

**DOI:** 10.21956/wellcomeopenres.20635.r54515

**Published:** 2023-02-03

**Authors:** Robert Simpson

**Affiliations:** 1 The New Zealand Institute of Plant and Food Research Limited, Palmerston North, New Zealand

This manuscript is a data note, briefly describing the assembly of the genome for the Noctuid setaceous Hebrew character. As a note it is necessarily brief, but it is sufficient to follow the methods used in sequencing and assembly and show the completeness of the submitted genome. *Xestia c-nigrum* is a cosmopolitan agricultural pest, continuing to extend its range and also a generalist feeder. This genome assembly will be an important tool both for those working directly on the insect, but also for those interested in generalist feeder and invasive species. There are no major problems with the note, but the following minor alterations should be considered.

1. There is more information about the mitochondrion in the abstract than in the body of the note. It would be helpful to add a short sentence stating exact size, and whether it is complete.
2. The reviewer understands the authors’ desire to include all material, but the second haplotype data is not well integrated with the remainder of the note. Either delete this or add more information. What amount of coverage? How much identity with the first haplotype? Any observed chromosome rearrangements?
3. If the second haplotype is included, the sentence beginning ‘Contigs corresponding to the second…’ should be amended to ‘Contigs corresponding to a second…’.

Are sufficient details of methods and materials provided to allow replication by others?

Yes

Is the rationale for creating the dataset(s) clearly described?

Yes

Are the datasets clearly presented in a useable and accessible format?

Yes

Are the protocols appropriate and is the work technically sound?

Yes

Reviewer Expertise:

Lepidopteran Genomics

I confirm that I have read this submission and believe that I have an appropriate level of expertise to confirm that it is of an acceptable scientific standard.

# Peer review: Reviewer response for version 1

**DOI:** 10.21956/wellcomeopenres.20635.r53624

**Published:** 2023-01-03

**Authors:** Vladimir A. Lukhtanov

**Affiliations:** 1 Department of Karyosystematics, Zoological Institute of Russian Academy of Sciences, St. Petersburg, Russian Federation

The authors present a chromosome-scale genome assembly for the setaceous Hebrew character, *Xestia c-nigrum. *The note provides brief information about the species, its genome, and the methods that were used to sequence and assemble the genome. This high-quality assembly will serve as a tool for a variety of studies as *Xestia c-nigrum* is an economically import species and frequently causes serious damage to agricultural crops. It is also important to note that it is an extremely widespread species across three continents: Asia, North America, and Europe. Therefore, as a result of the presented study, scientists from many countries have received free access to the necessary and useful genetic resource. The presented note will serve as a starting point for all these future studies.

 I have the following minor corrections and suggestions:

1. In the title “Xestia c-nigrum, (Linnaeus, 1758)”, remove the comma after c-nigrum.
2. The collection locality and coordinates are repeated twice in the note.
3. A second specimen of *Xestia c-nigrum *is mentioned, but it is not relevant, as it was not involved in obtaining DNA and sequencing.
4. In the article, it would be valuable to mention the data on the karyotype of *Xestia c-nigrum* obtained earlier using the methods of classical cytogenetics. The haploid number of chromosomes for this species was first given by Bigger as n=29 for a population from Great Britain (Bigger, 1961 ^1^, the species was identified as *Amathes c-nigrum*). Apparently, this count of the number of chromosomes was incorrect. It was corrected by Werner (1975 ^2^), who identified the haploid number n=31 for the population from Germany.

Are sufficient details of methods and materials provided to allow replication by others?

Yes

Is the rationale for creating the dataset(s) clearly described?

Yes

Are the datasets clearly presented in a useable and accessible format?

Yes

Are the protocols appropriate and is the work technically sound?

Yes

Reviewer Expertise:

Cytogenetics, genomics, Lepidoptera taxonomy

I confirm that I have read this submission and believe that I have an appropriate level of expertise to confirm that it is of an acceptable scientific standard.

## References

[1] : Chromosome numbers of Lepidoptera. Part II. *Entomologist’s Gaz.* .1961;12:85-89

[2] : Zur Evolution des Karyotyps bei den Lepidopteren. Die Chromosomenzahlen der Noctuiden. *Genetica* .1975;45(3) : 10.1007/BF01508313 377-395 10.1007/BF01508313
